# The Construction of Cucurbit[7]uril-Based Supramolecular Nanomedicine for Glioma Therapy

**DOI:** 10.3389/fchem.2022.867815

**Published:** 2022-03-16

**Authors:** Mantao Chen, Chi Hu, Shengxiang Zhang, Dan Wu, Zhengwei Mao, Xiujue Zheng

**Affiliations:** ^1^ Department of Neurosurgery, First Affiliated Hospital, School of Medicine, Zhejiang University, Hangzhou, China; ^2^ College of Materials Science and Engineering, Zhejiang University of Technology, Hangzhou, China; ^3^ MOE Key Laboratory of Macromolecular Synthesis and Functionalization, Department of Polymer Science and Engineering, Zhejiang University, Hangzhou, China

**Keywords:** supramolecular chemistry, nanomedicine, cucurbit[7]uril, glioma, chemotherapy

## Abstract

Two supramolecular nanomedicines (CB[7]⊃DOX and CB[7]⊃CPT) based on the host–guest recognition between CB[7] and anticancer drugs were constructed. After supramolecular modification, the stability and water solubility of DOX and CPT were greatly improved, and the anticancer activities of chemotherapeutic drugs were effectively maintained. This work provided a simple but efficient method to enrich supramolecular nanomedicines for cancer therapy.

## Introduction

Being able to reach every corners of the body, chemotherapy is the first choice for the patients diagnosed with metastatic cancers. Chemotherapy can suppress the fast proliferation of tumor cells, yet they also restrain the rapid growth of the bone marrow, hair follicles, and gastrointestinal tract cells (Chabner and Roberts, 2005; Pérez-Herrero and Fernández-Medarde, 2015; Wu Dan et al., 2021). Hence, severe adverse reactions are always the undesired appurtenances of cancer chemotherapy. Because most of the chemotherapeutic drugs are hydrophobic molecules, they have poor solubility and stability in physiological environments, thus leading to the limited therapeutic effect (Zhou et al., 2017). Nanomedicines are receiving increasing attentions over the past decades because of their ability to promote the pharmacokinetics of drugs, enhance the therapeutic efficacy, and decrease the side effects of drugs (Janib et al., 2010; Lee et al., 2012; Oun et al., 2018; Xue et al., 2018; Wu et al., 2022). Incorporation of traditional chemotherapeutic drugs into nanomedicine is an effective method to overcome the limitations of conventional chemotherapy.

Nanomedicines constructed based on supramolecular chemistry are preferred for their feasibility of preparation, biodegradability, and stimuli responsiveness. Supramolecular chemistry, chemistry that is beyond the molecule, is based on various non-covalent interactions, such as hydrogen bonding, charge-transfer interactions, *π*–*π* stacking interactions, electrostatic interactions, and host–guest interactions (Lehn, 1988; Erbas-Cakmak et al., 2015; Liu et al., 2015; Xue et al., 2015; You et al., 2015). Supramolecular systems self-assembled from host–guest complexation exhibit outstanding properties owing to the introduction of host molecules, showing promising potentials in biomedical applications (Yu et al., 2012; Yu et al., 2013a; Webber and Langer, 2017). The complexation between hosts and guests can be regulated by multiple external stimuli, such as ions, temperature, redox, pH, light, and enzyme (Harada, 2001; Yan et al., 2012; Cook et al., 2013; Yu et al., 2013b; Spa et al., 2018). Fortunately, some differences in the biological microenvironment between normal and tumor cells can also be used to adjust the binding affinities of host–guest reactions, realizing precise cancer theranostics (Laza-Knoerr et al., 2010; Tiwari et al., 2012; Cafeo et al., 2013; Tibbitt et al., 2016; Yu et al., 2018). Macrocylic hosts including crown ethers, cyclodextrins, calixarenes, pillararenes, and cucurbiturils usually own hydrophobic cavities which can be used to embed guests (Kim et al., 2007; Niu et al., 2011; Appel et al., 2012; Yu et al., 2015). Cucurbit[*n*]urils (CB[*n*]s, *n* = 5–8, 13–15) are pumpkin-liked macrocylic host in which the glycoluril units and methylene bridges are repeatedly linked (Lee et al., 2003; Lagona et al., 2005; Ni et al., 2014; Li et al., 2018; Xu et al., 2018). Unlike cyclodextrins whose driving force are hydrophobic interaction possessing moderate binding affinity in the range of 10^2^–10^4^ M, the binding affinities of CB[*n*]s are much higher mainly arising from the cooperation of hydrophobic interactions and ion–dipole interactions. Due to the difference in polarity and cavity sizes, different unique host−guest recognitions are built between CB[*n*]s and different guests. Considering their excellent biocompatibility and outstanding molecular recognitions, CB[*n*]s have been extensively employed to fabricate drug delivery systems for disease theranostics (Sun et al., 2018; Ding et al., 2019; Wu Han et al., 2021).

Herein, we constructed two supramolecular nanomedicines (CB[7]⊃DOX and CB[7]⊃CPT) based on the host–guest recognition between CB[7] and two anticancer drugs (doxorubicin (DOX) and camptothecin (CPT)), mainly driven by host−guest interactions. After supramolecular modification, the stability and water solubility of supramolecular nanomedicines were greatly improved, and the anticancer activities of DOX and CPT were effectively maintained. Attributing to the simplicity and feasibility of preparation as well as the good therapeutic effect, two supramolecular nanomedicines have great potentials to realize clinical transformation in the near future.

## Results and Discussion

### Investigation of the Host–Guest Complexation Between CB[7] and 3-Methylcyclohexylamine

**FIGURE 1:**
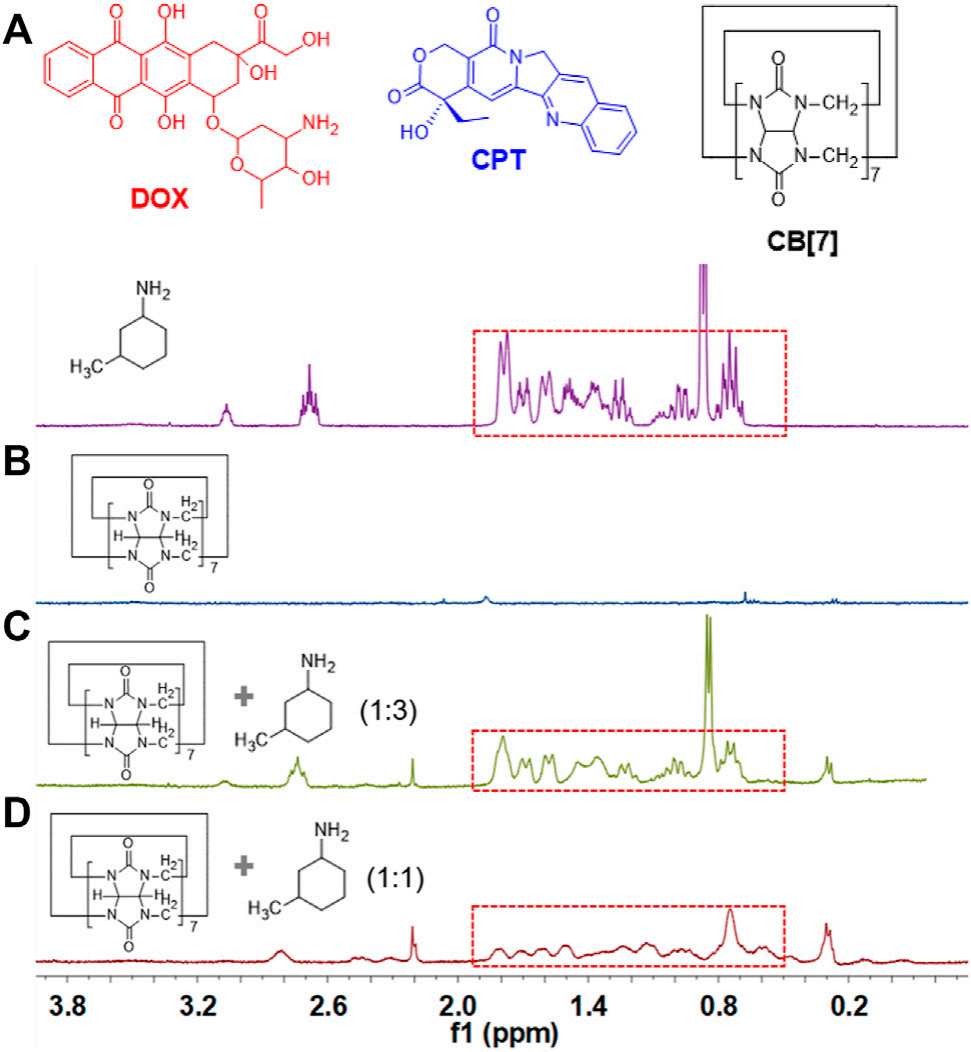
Chemical structure of DOX, CPT, and CB[7]. Partial ^1^H NMR spectra (D_2_O, room temperature, 400 MHz): **(A)** 3-methylcyclohexylamine (isomer form), **(B)** CB[7], **(C)** CB[7] and 3-methylcyclohexylamine (molar ratio: 1 : 3), and **(D)** CB[7] and 3-methylcyclohexylamine (molar ratio: 1: 1).

^1^H NMR spectroscopy was utilized to study the host–guest recognition between CB[7] and DOX. Because DOX is insoluble in aqueous solution, 3-methylcyclohexylamine was used as a model guest. As shown in Figure 1D, when equimolar amounts of CB[7] and 3-methylcyclohexylamine were mixed in D_2_O, obvious chemical shift changes of the protons on 3-methylcyclohexylamine were observed, suggesting that 3-methylcyclohexylamine was encapsulated in the hydrophobic cavity of CB[7]. When three equivalents of 3-methylcyclohexylamine were added into CB[7], the peak shape of 3-methylcyclohexylamine became similar to that of free 3-methylcyclohexylamine (Figure 1C), suggesting that there were excess free 3-methylcyclohexylamine in solution. On the other hand, nuclear Overhauser effect correlation signals between CB[7] and 3-methylcyclohexylamine were observed (Supplementary Figure S2), further demonstrating host–guest complexation occurred between CB[7] and 3-methylcyclohexylamine, in which the guest molecular deeply penetrated into the cavity of CB[7].

Isothermal titration calorimetry (ITC) was used to acquire the thermodynamic information for the complexation between CB[7] and 3-methylcyclohexylamine. As shown in Supplementary Figure S3, the *K*_*a*_ values of CB[7]⊃3-methylcyclohexylamine were determined to be (2.73 ± 0.84) × 10^6^ M^−1^, indicating the binding affinity was very high and was favorable for the fabrication of supramolecular systems in physiological environments. Furthermore, the enthalpy changes (Δ*H* < 0) indicated that the host–guest recognition between CB[7] and 3-methylcyclohexylamine was driven by enthalpy changes. All these results indicated that the complexation between CB[7] and DOX could take place *via* host–guest interactions, which paved the way for the construction of supramolecular nanomedicine.

### Investigation of the Morphology of Supramolecular Nanomedicines

**FIGURE 2:**
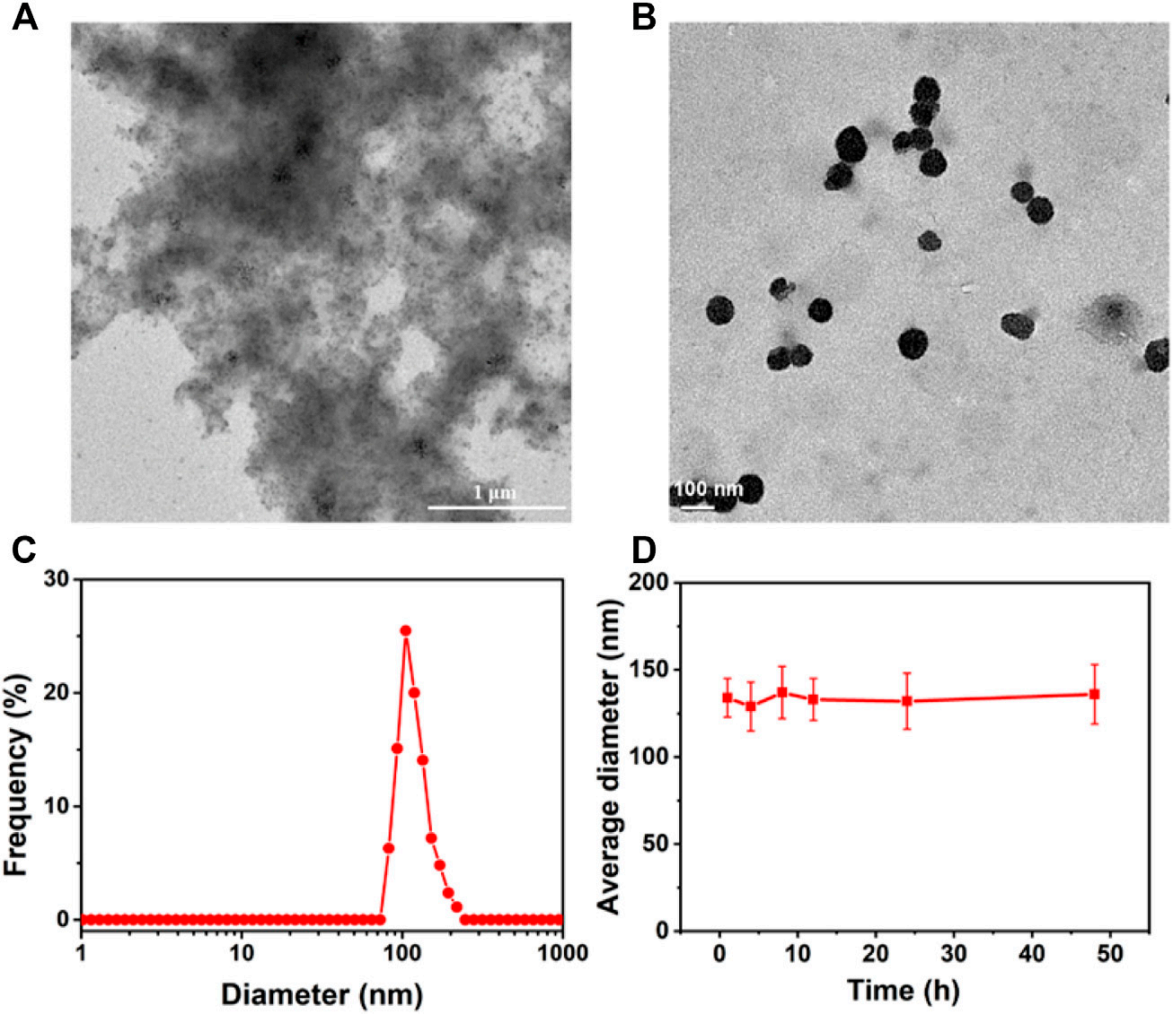
TEM images of aggregates formed from DOX **(A)** and spherical nanoparticles formed from CB[7]⊃DOX **(B)**, **(C)** DLS size distributions of nanoparticles self-assembled from CB[7]⊃DOX, and **(D)** diameter changes of nanoparticles formed from CB[7]⊃DOX after incubation in PBS for different times.

**FIGURE 4:**
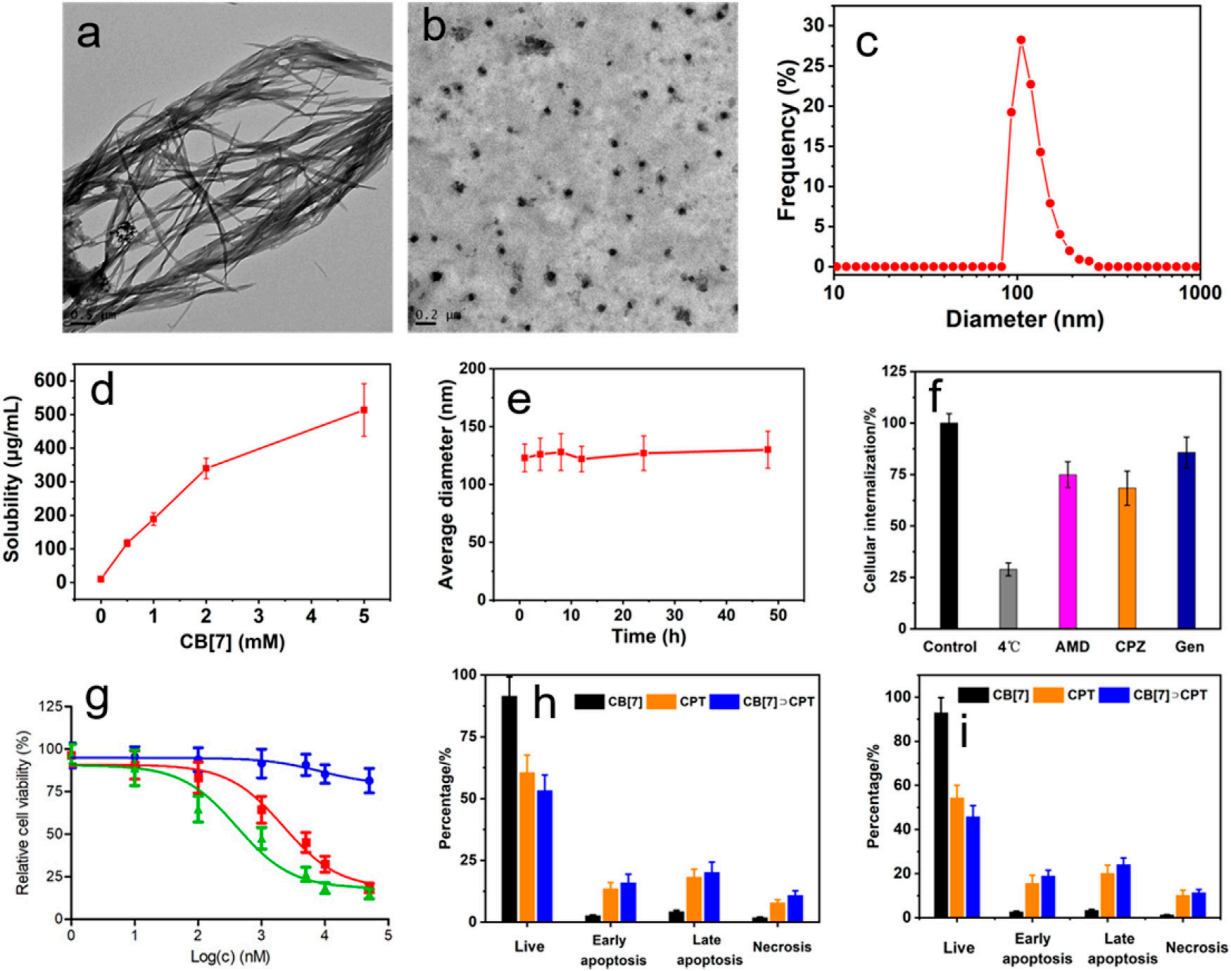
TEM images of aggregates formed from CPT **(A)** and spherical nanoparticles formed from CB[7]⊃CPT **(B)**, **(C)** DLS size distributions of nanoparticles self-assembled from CB[7]⊃CPT, **(D)** solubility improvement of CPT after the addition of different quantities of CB[7], **(E)** average diameter change of CB[7]⊃CPT after incubation in PBS for different times, **(F)** internalization efficiency analysis of CB[7]⊃CPT after incubation with different endocytosis inhibitors, **(G)** cytotoxicity against U87 cells incubated with different concentrations of CB[7]⊃CPT for 24 h (blue: CB[7]; red: CPT; green: CB[7]⊃CPT), and flow cytometric analysis of Annexin-V/PI dual-staining of U87 cells **(H)** and HeLa cells **(I)** after different treatments.

After confirming the possible inclusion complexation between CB[7] and DOX, we studied the morphology of supramolecular nanomedicines in water. As can be seen in Figure 2A, large precipitates were observed in the DOX group owing to the low solubility of DOX in water, but regular spherical nanoparticles with a diameter of about 100 nm were observed in the presence of CB[7] (Figure 2B), suggesting hydrophilic CB[7] significantly inhibited the *π*–π stacking and improved the water solubility of DOX. The average diameter of CB[7]⊃DOX measured by the dynamic light scattering (DLS) experiment was 121 ± 13.4 nm (Figure 2C), which is in accordance with the result from transmission electron microscopy (TEM). The average diameter of the supramolecular nanomedicine almost remained unchanged after incubation in PBS for 48 h (Figures 2D, 4E), implying that the stability of supramolecular nanomedicine was good in the physiological environment.

### Investigation of the Internalization Behavior of Supramolecular Nanomedicines

**FIGURE 3:**
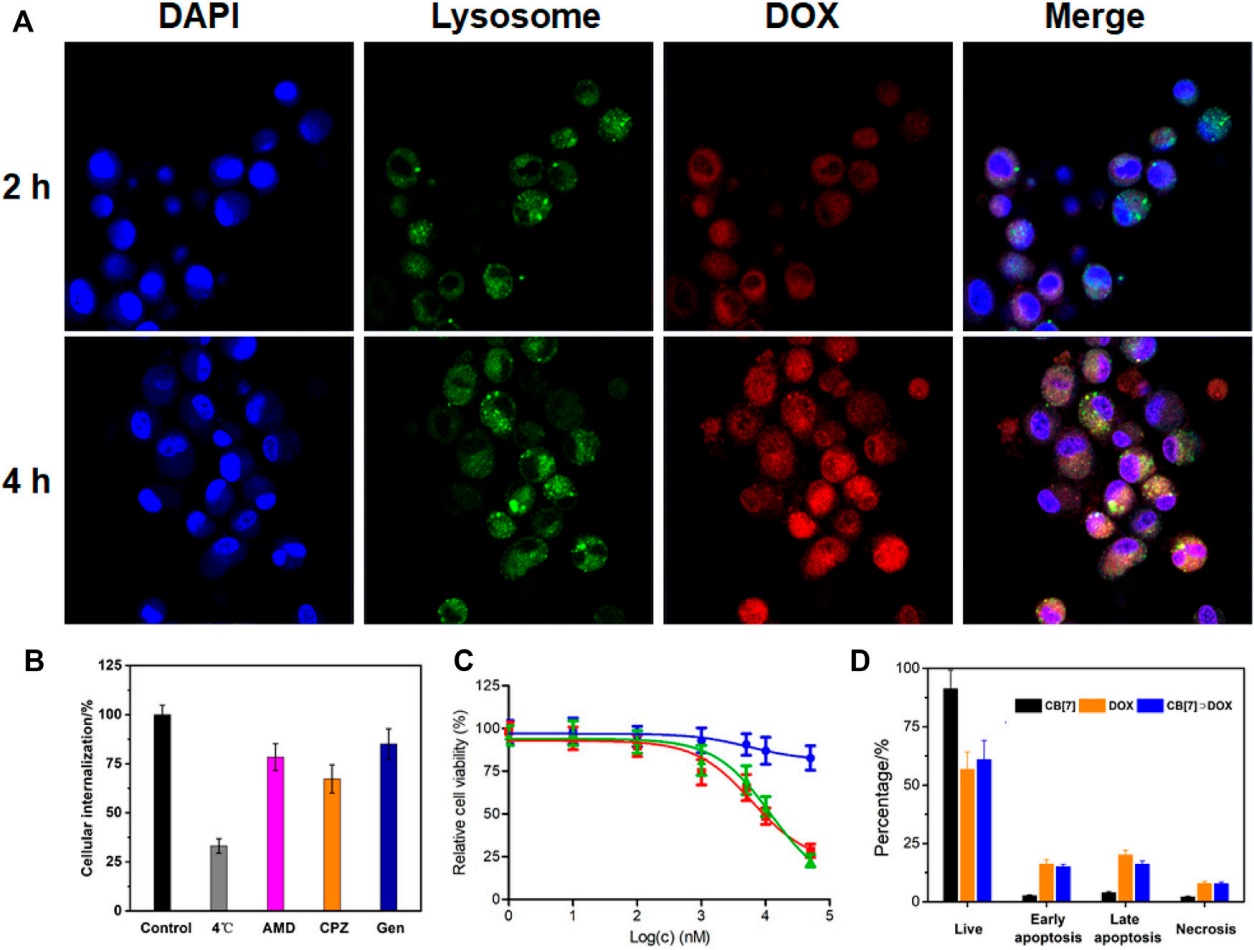
**(A)** CLSM images of HeLa cells incubated with CB[7]⊃DOX for different time periods, **(B)** internalization efficiency analysis of CB[7]⊃DOX after incubation with different endocytosis inhibitors, **(C)** cytotoxicity against HeLa cells incubated with different concentrations of CB[7]⊃DOX for 24 h (blue: CB[7]; red: DOX; green: CB[7]⊃DOX), and **(D)** flow cytometric analysis of Annexin-V/PI dual-staining of HeLa cells after different treatments.

The internalization behavior of CB[7]⊃DOX was then studied by confocal laser scanning microscopy (CLSM). As shown in Figure 3A, obvious red fluorescence arising from DOX was observed in the cytoplasm after 2 h incubation, proving that CB[7]⊃DOX was easily internalized by HeLa cells. When incubation time reached 4 h, the red fluorescence appeared in both the cytoplasm and nucleus, suggesting that CB[7]⊃DOX could enter into the nucleus to prime their therapeutic actions. The endocytic pathways of supramolecular nanomedicines were evaluated by adding different endocytosis inhibitors, such as amiloride-HCl (AMD), chlorpromazine (CPZ), and genistein (Gen). As shown in Figures 3B, 4F, the internalization of supramolecular nanomedicines was greatly inhibited at 4°C, indicating their cell uptake was energy-dependent. Meanwhile, pre-treatment with CPZ, AMD, or Gen led to the difference in decrease of cellular uptakes, suggesting that the endocytosis of nanomedicines was mediated by the cooperation of clathrin-, micropinocytosis-, and caveolae-participated endocytic pathways.

### Investigation of Anticancer Efficacy of Supramolecular Nanomedicines

The therapeutic efficacy of supramolecular nanomedicines against U87 and HeLa cells was assessed by a 3-(4′,5′-dimethylthiazol-2′-yl)-2,5-diphenyltetrazolium bromide (MTT) assay. The IC_50_ values of CB[7]⊃DOX against U87 and HeLa cells were 12.5 ± 1.43 and 11.9 ± 1.24 μM, respectively, which were comparable to the IC_50_ values of DOX (6.52 ± 0.70 and 5.47 ± 0.68 μM against U87 and HeLa cells, respectively) (Figure 3C and Supplementary Figure S4). An Annexin V-FITC/propidium iodide (PI) dual-staining assay was utilized to analyze the percentage of apoptotic cells. Figure 4H showed that a large percentage of the apoptotic (31.3%) and necrotic (7.8%) cells was monitored for the HeLa cells treated with CB[7]⊃DOX, which was similar to the values for the cells treated with free DOX. CB[7]⊃DOX also showed a similar ability to induce the apoptosis of U87 cells (Supplementary Figure S6), further demonstrating the anticancer efficacy of DOX was fully kept after supramolecular fabrication. The percentage of apoptotic cells induced by CB[7]⊃DOX was also higher than that in the free DOX group (Figure 3D and Supplementary Figure S6), suggesting the anticancer activity of DOX was highly maintained.

Apart from DOX, CB[7] could also be used as a host to interact with CPT through molecular recognition. The host−guest interaction between CB[7] and CPT was verified by biolayer interferometry (Supplementary Figure S7), which demonstrated that CPT could be stably encapsulated by CB[7] due to the high binding affinity. More interestingly, the host−guest complexation was able to regulate the self-assembly of CPT. Due to the severe *π*–π stacking interactions, CPT formed large aggregates in aqueous solution (Figure 4A). By the formation of the host−guest inclusion complex, CB[7]⊃CPT self-assembled into nanoparticles (Figure 4B). The average diameter of the obtained nanoparticles was measured to be 133 ± 17.2 nm by DLS (Figure 4C). The solubility of CPT is extremely poor, which was determined to be 10.3 ± 0.87 μg/ml in PBS. It should be emphasized that the solubility of CPT greatly increased in the presence of CB[7] (Figure 4D) because water soluble CB[7] encapsulated CPT and significantly inhibited the *π*–π stacking of CPT. Additionally, the stability of the nanoparticles formed by CB[7]⊃CPT was satisfactory, and negligible changes in the size of the assemblies were detected by DLS over 48 h (Figure 4E). Similar to CB[7]⊃DOX, the endocytosis of nanoparticles assembled from CB[7]⊃CPT was also mediated by the cooperation of clathrin-, micropinocytosis-, and caveolae-participated endocytic pathways. The anticancer capability of CB[7]⊃CPT was evaluated by an MTT assay, which indicated that the IC_50_ value of CB[7]⊃CPT was lower than that of free CPT. The possible reason was that the aggregates formed from free CPT were unfavorable for cellular internalization, while the supramolecular modification optimized the size of assemblies prepared from CB[7]⊃CPT, thus enhancing cell uptake. Annexin V-FITC/PI dual staining further demonstrated that the therapeutic efficacy of CPT was fully maintained after supramolecular fabrication (Figures 4H,I).

## Conclusion

In summary, two supramolecular nanomedicines were developed based on the host–guest recognition motif, in which CB[7] acted as the host, and anticancer drugs CPT and DOX acted as the guests. In the aqueous solution, CB[7]⊃CPT and CB[7]⊃DOX self-assembled into spherical nanoparticles with the diameter of around 100 nm. Attributed to the water-soluble CB[7], the stability and solubility of CPT and DOX were significantly improved. CLSM experiments showed that both supramolecular nanomedicines could be efficiently internalized and enter into the nucleus of tumor cells. MTT and Annexin V-FITC/PI dual-staining experiments demonstrated that two supramolecular nanomedicines could efficiently induce apoptosis of U87 cells and showed a good anticancer effect toward glioma. The current study provides a simple but a high-efficiency supramolecular method to improve the performance of traditional small molecular anticancer drugs, making a contribution for the preclinical drugs to realize clinical transformation.

## Data Availability

The raw data supporting the conclusions of this article will be made available by the authors, without undue reservation.
