# Supplementary material for: The Construction of Cucurbit[7]uril-Based Supramolecular Nanomedicine for Glioma Therapy
Source: Front Chem. 2022 Mar 16;10:867815. doi: 10.3389/fchem.2022.867815 (PMC8966231; doi:10.3389/fchem.2022.867815)
Supplement: Supplementary file 1 [file DataSheet1.docx]

**Supporting information**

**The Construction of Cucurbit[7]uril-based Supramolecular Nanomedicines for Glioma Therapy**

Mantao Chen^1#^, Chi Hu^1#^, Shengxiang Zhang^1^, Dan Wu^2^*, Zhengwei Mao^3^*, Xiujue Zheng^1^*

^1^ Department of Neurosurgery, First Affiliated Hospital, School of Medicine, Zhejiang University, No. 79, Qingchun Road, Hangzhou, Zhejiang Province, China.

^2^ College of Materials Science and Engineering, Zhejiang University of Technology, Hangzhou, 310014, P. R. China.

^3^ MOE Key Laboratory of Macromolecular Synthesis and Functionalization, Department of Polymer Science and Engineering, Zhejiang University, Hangzhou 310027, P. R. China

*** Correspondence:**

Xiujue Zheng, zxj8065@zju.edu.cn
Dan Wu, [danwu@zjut.edu.cn](mailto:danwu@zjut.edu.cn)

Zhengwei Mao, zwmao@zju.edu.cn

# These authors contribute equally

**Materials and Methods.** Cucurbit[7]uril (CB[7]), 3-methylcyclohexylamine (isomer form), doxorubicin (DOX) and camptothecin (CPT) were purchased from Aladdin or Sigma-Aldrich. Solvents were either employed as purchased or dried according to procedures described in the literature. Millipore ultrapure water was obtained on a Milli-Q purification system. Transmission electron microscopy (TEM) investigations were carried out on a HT-7700 instrument. Confocal laser scanning microscopy (CLSM) images was recorded on a LSM710META (Zeiss) microscope. ^1^H NMR spectra were recorded on a Bruker Avance 400 spectrometer. The sizes of the nanoformulations were determined by a DLS analyzer (Zetasizer Nano ZS90 Malvern Instruments, Malvern). ITC experiments were carried out with a Microcal VP-ITC calorimeter at 298.1 K.

**TEM studies.** The morphology of free drugs and host−guest complexes was characterized by TEM. Free drug (CPT or DOX) was solubilized in DMSO (0.200 mmol), the solution (0.1 mL) was injected into distilled water (2 mL) or PBS (2 mL) under sonication. For CB[7]⊃DOX, CB[7] and DOX (molar ration is 2:1) were dissolved in DMSO and the solution was stirred for 1 h at room temperature. The concentration of CPT was controlled as 0.500 mM in this stocking solution. The solution (0.1 mL) was injected into distilled water (2 mL) or PBS (2 mL) under sonication. For CB[7]⊃CPT, CB[7] and CPT (molar ration is 5:1) were dissolved in DMSO and the solution was stirred for 2 h at room temperature. The concentration of CPT was controlled as 0.100 mM in this stocking solution. The solution (0.1 mL) was injected into distilled water (2 mL) or PBS (2 mL) under sonication. TEM samples were prepared by drop-coating a solution onto a carbon-coated copper grid.

**Determination the solubility of CPT.** The solubility of CPT was determined in the absence and presence of CB[7]. Briefly, CPT (0.100 g) was dispersed in aqueous solution with/without CB[7] at different concentration, and the mixture was warmed to 80 ^o^C under sonication for 1 h. The mixture was cooled down, and the undissolved CPT was obtained by centrifugation. After drying under vacuum at 50 ^o^C overnight, the mass of undissolved CPT was measured. Accordingly, the dissolved CPT could be calculated.

**Cell Cultures.** HeLa cells was cultured in Dulbecco's modified Eagle's medium (DMEM) containing 10% fetal bovine serum (FBS) and 1% penicillin/streptomycin. U87MG cells were incubated in Minimum Essential Medium (MEM) containing FBS (10%) and penicillin/streptomycin (1%). Cells grew as a monolayer and were detached upon confluence using trypsin (0.5% *w*/*v* in PBS). The cells were harvested from the cell culture medium by incubating in a trypsin solution for 5 min. The cells were centrifuged, and the supernatant was discarded. A 3 mL portion of serum-supplemented DMEM was added to neutralize any residual trypsin. The cells were resuspended in serum-supplemented DMEM at a concentration of 1 × 10^4^ cells/mL. Cells were cultured at 37 °C and 5% CO_2_.

**Evaluation of Cytotoxicity.** The cytotoxicity of CPT, DOX, CB[7]⊃CPT and CB[7]⊃DOX against HeLa and U87 cells was determined by 3-(4′,5′-dimethylthiazol-2′-yl)-2,5-diphenyl tetrazolium bromide (MTT) assays in a 96-well cell culture plate. All solutions were sterilized by filtration with a 0.22 μm filter before tests. HeLa and U87 cells were seeded at a density of 1 × 10^4^ cells/well in a 96-well plate, and incubated for 24 h for attachment. Cells were then incubated with free CPT, DOX, CB[7]⊃CPT and CB[7]⊃DOX at various concentrations for 24 h. After washing the cells with PBS buffer, 20 μL of a MTT solution (5 mg/mL) were added to each well. After 4 h of incubation at 37 °C, the MTT solution was removed, and the insoluble formazan crystals that formed were dissolved in 100 μL of dimethylsulfoxide (DMSO). The absorbance of the formazan product was measured at 570 nm using a spectrophotometer (Bio-Rad Model 680). Untreated cells in media were used as a control. All experiments were carried out with five replicates.

**Evaluations of Endocytosis Pathways.** The inhibition studies of endocytosis were performed as follows. For the inhibition of energy-dependent endocytosis, HeLa cells were incubated with the nanoparticles self-assembled from CB[7]⊃DOX at 4 ^o^C. For the inhibition of caveolae-mediated uptake, cells were pre-incubated with genistein (Gen, 1.00 mM) for 30 min at 37 ^o^C/5% CO_2_. The media were then changed to fresh media containing CB[7]⊃DOX and further incubated for 4 h at 37 ^o^C/5% CO_2_. For the inhibition of macropinocytosis, cells were pre-incubated in serum-free DMEM with the amiloride (AMD, 1.00 mM) for 30 min at 37 ^o^C/5% CO_2_, then treated with CB[7]⊃DOX for 4 h. For the inhibition of clathrin-mediated endocytosis, cells were pretreated with chlorpromazine (CPZ, 10 µg/mL) for 30 min at 37 ^o^C/5% CO_2_ and then treated with CB[7]⊃DOX for 4 h. For all endocytic inhibition tests, after exposure to the respective inhibitors and CB[7]⊃DOX, the cells were washed with cold PBS and followed by quantifying the fluorescence intensity under CLSM. Percent internalization was normalized to particle internalization in the absence of inhibitors. Data are mean values (200 cells, three experiments).

***In Vitro* Cell Accumulation Determined** **by CLSM.** HeLa cells were treated with CB[7]⊃DOX (the concentration of DOX was 5.00 μM) in the culture medium at 37 °C for 2 h, and 4 h, respectively. The cells were washed three times with PBS, fixed with fresh 4.0% formaldehyde at room temperature for 15 min. After washing with PBS for three times, the cells were stained with DAPI and Lysotracker Green for 30 min, and washed with PBS for two times. The images were taken using a LSM710META confocal laser scanning microscope (ZEISS).

**Flow Cytometry for Apoptosis Assay.** HeLa and U87 cells were seeded in 6-well plates at a density of 3 × 10^5^ cells/well and incubated in culture medium for 24 h at 37 °C. After that, the culture medium containing free drug (DOX or CPT) or the complex (CB[7]⊃CPT or CB[7]⊃DOX) was added. The concentration of DOX and CPT in these formulations was 5.00 mM and 1.00 mM, respectively. After 24 h incubation, the cells were thoroughly washed with PBS and harvested with typsin. The collected cells were washed three times with PBS and resuspended in 0.3 mL of PBS. The cells were stained with Annexin V-FITC and propidium iodide according to the protocol. The cells were incubated for 15 min at room temperature in the dark and added with 400 μL of 1× binding buffer. The stained cells were analyzed by flow cytometry. A total of 1 × 10^4^ events were counted for analysis.

**Statistical Analysis.** Data are expressed as mean standard deviation (SD). Analysis of variance (ANOVA), followed by Student’s t-test, was used to determine the significant differences among the groups (***P* < 0.01, and ****P* < 0.001).


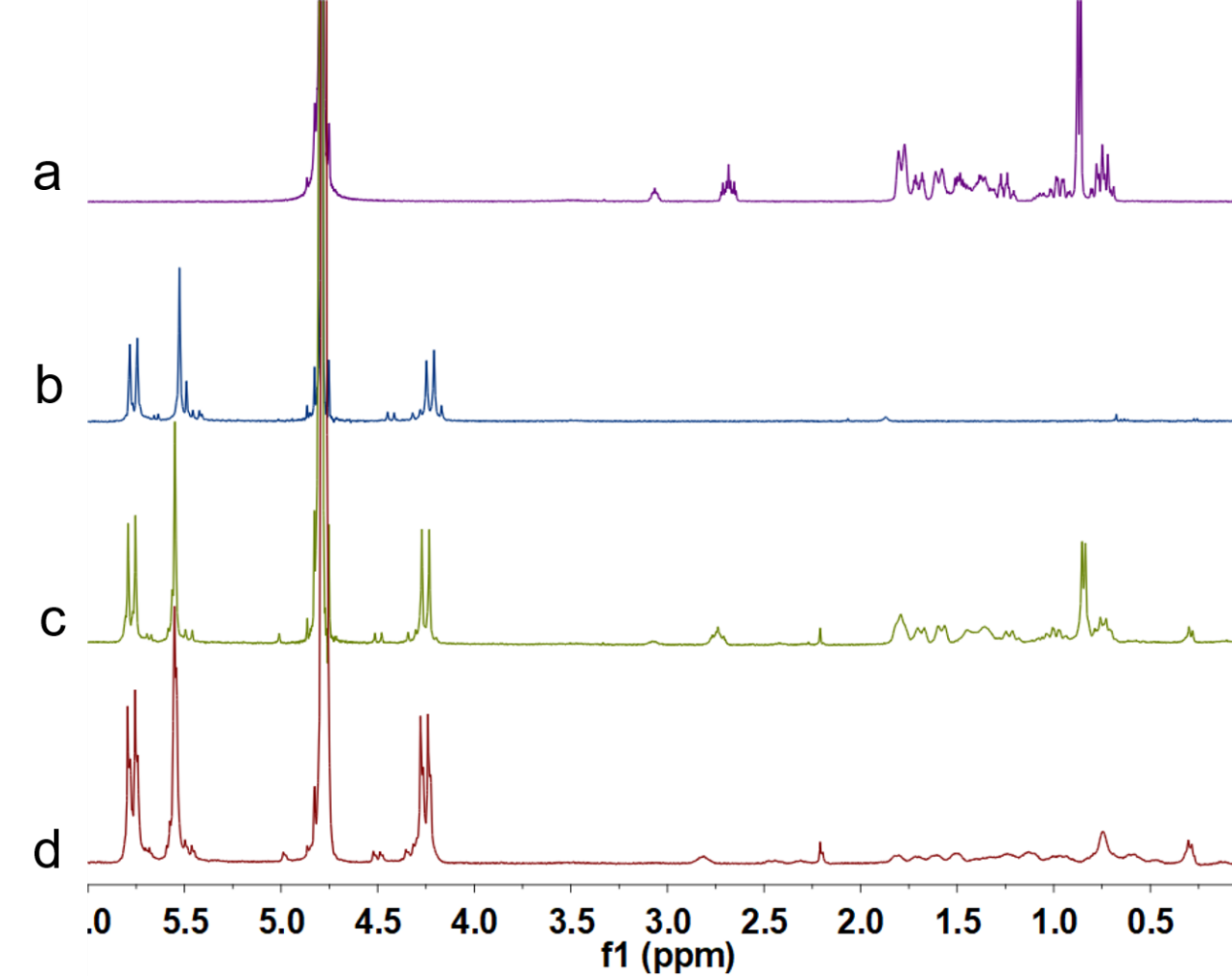


**Fig. S1** Partial ^1^H NMR spectra (D_2_O, room temperature, 400 MHz): (a) 3-methylcyclohexylamine; (b) CB[7]; (c) CB[7] and 3-methylcyclohexylamine (molar ratio: 1 : 3); (d) CB[7] and 3-methylcyclohexylamine (molar ratio: 1 : 1).


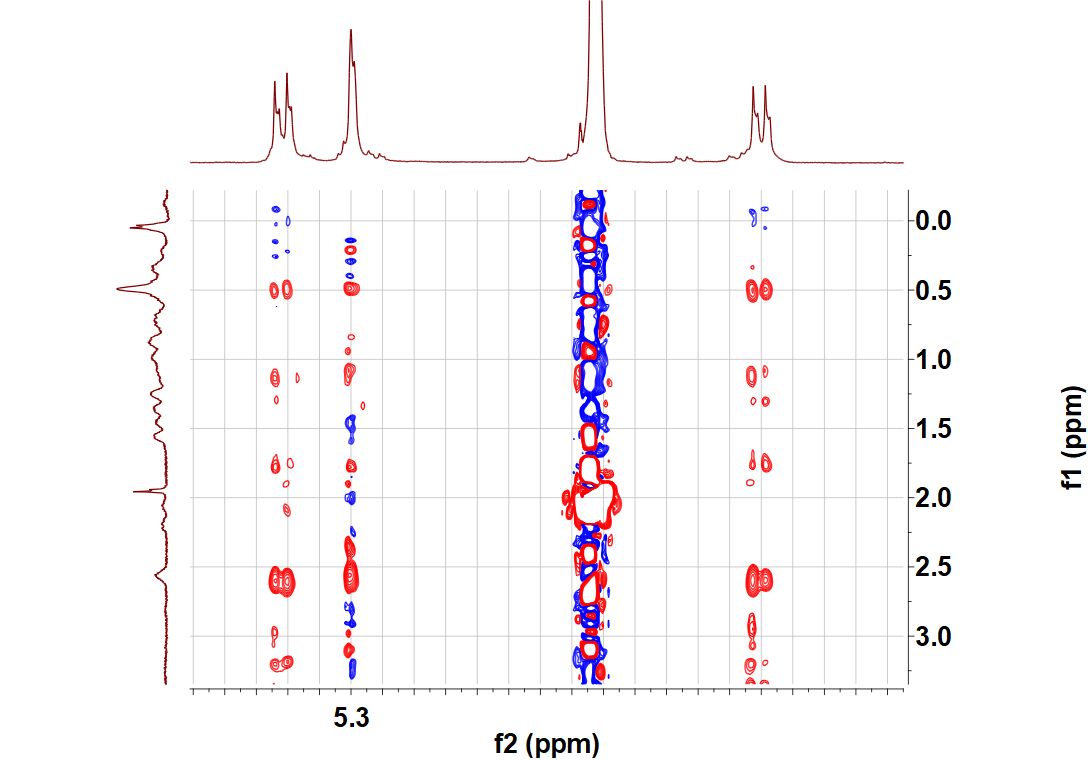


**Figure S2.** Partial NOESY NMR spectra (600 MHz, D_2_O, room temperature) of CB[7] and 3-methylcyclohexylamine.


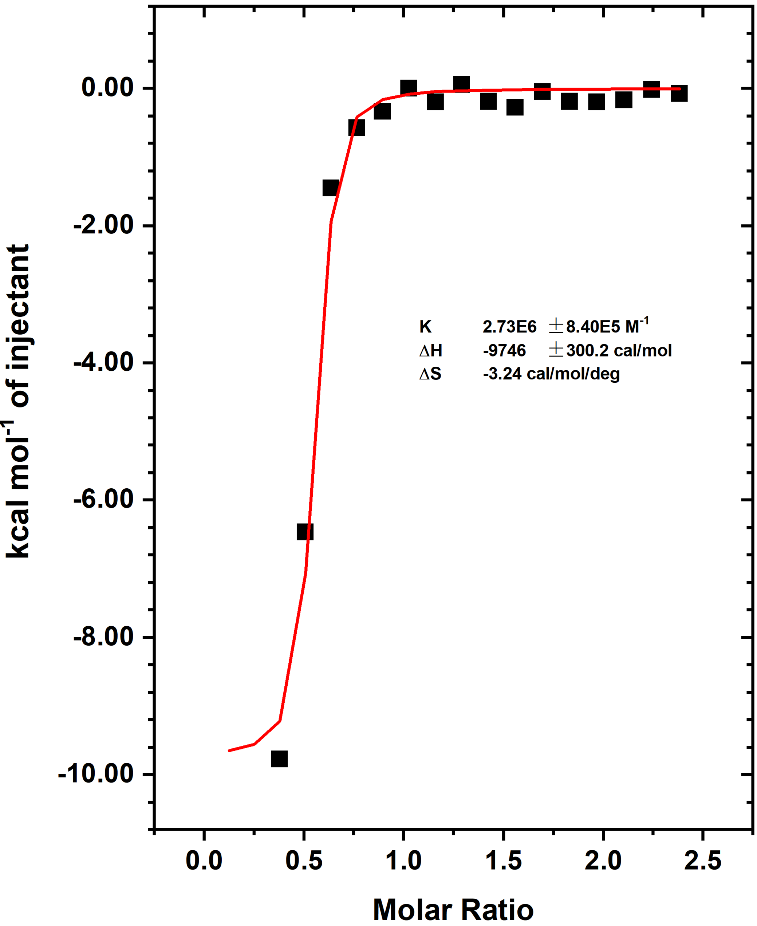


**Figure S3.** Microcalorimetric titration of CB[7] (2.00 mM, 10 µL per injection) with 3-methylcyclohexylamine (0.100 mM) in water at 298.15 K.


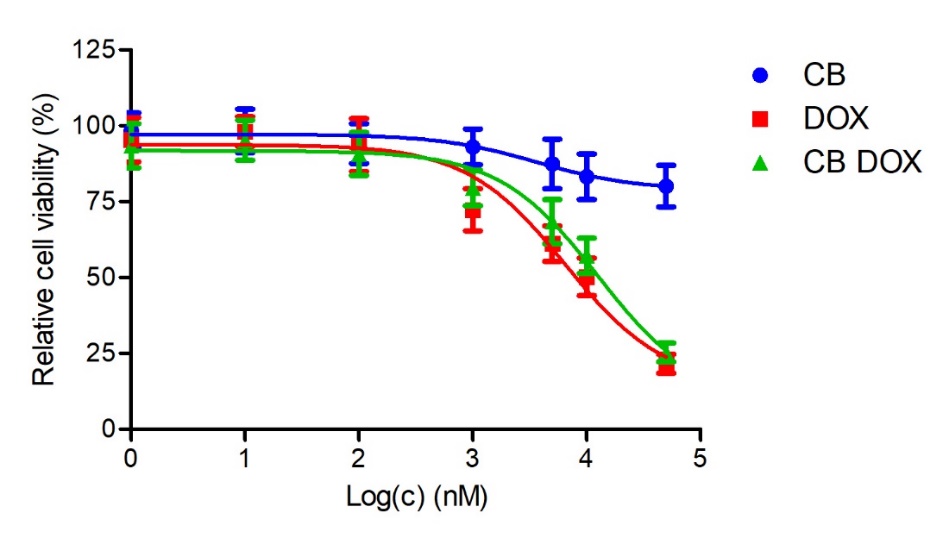


**Figure S4.** Cytotoxicity against U87 cells incubated with different concentrations of CB[7]⊃DOX for 24 h (blue: CB[7]; red: DOX; green: CB[7]⊃DOX).


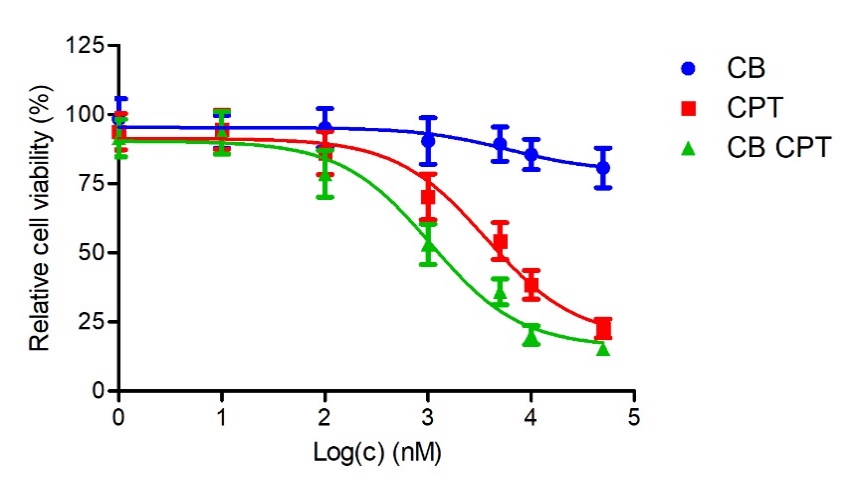


**Figure S5.** Cytotoxicity against HeLa cells incubated with different concentrations of CB[7]⊃CPT for 24 h (blue: CB[7], red: CPT, green: CB[7]⊃CPT).


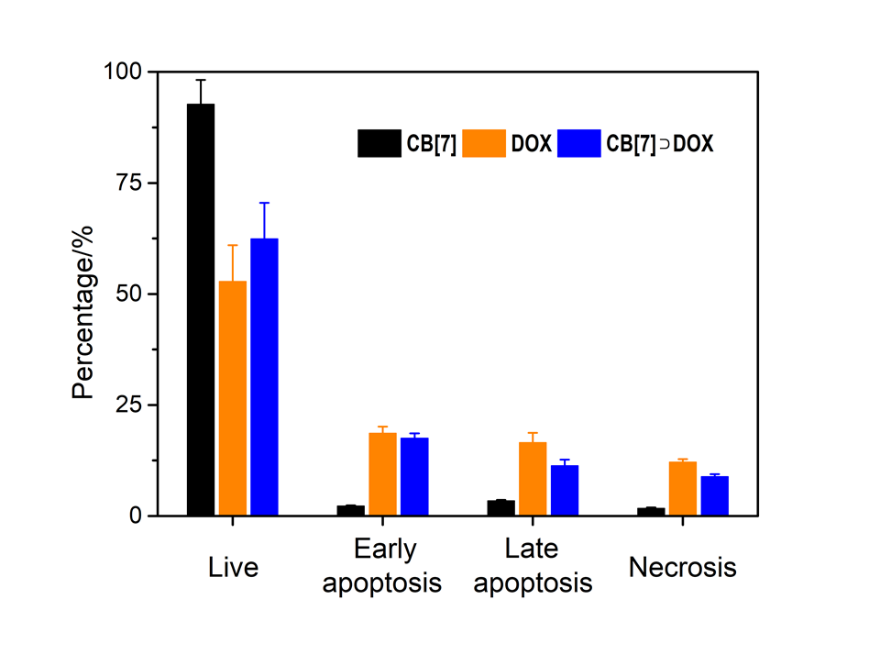


**Figure S6.** Flow cytometric analysis of Annexin-V/PI dual-staining of U87 cells after different treatments.


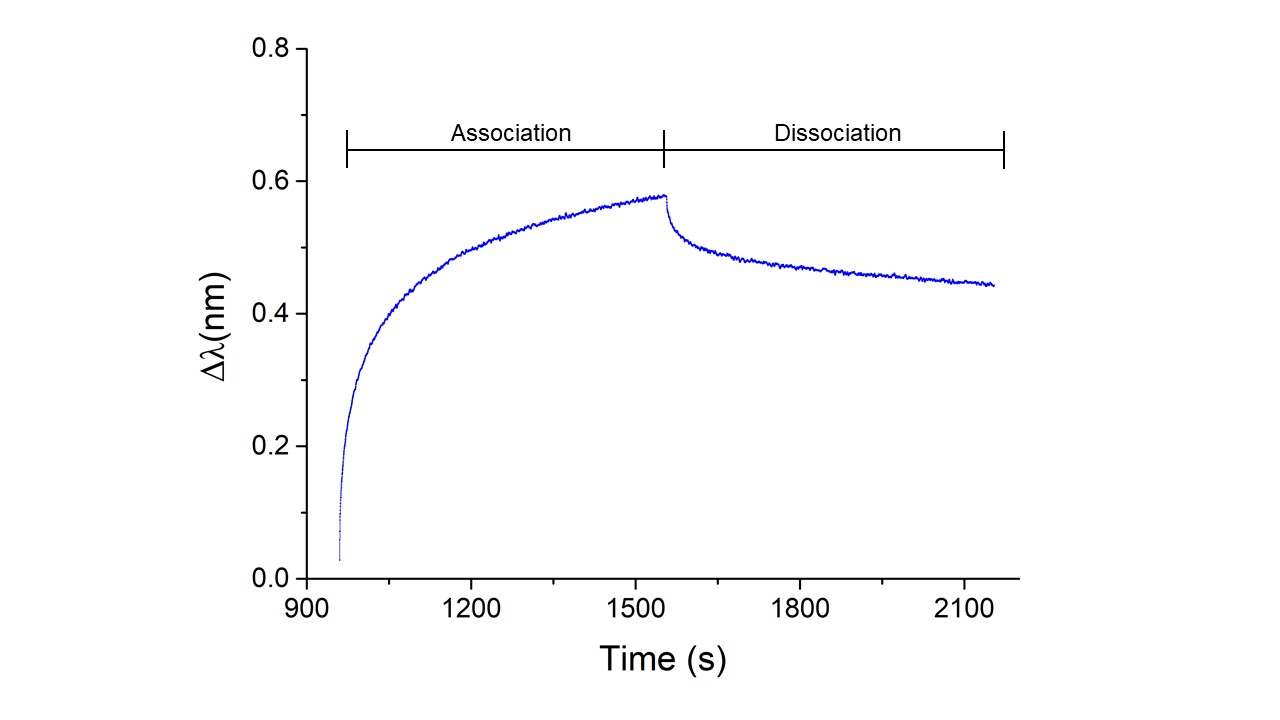


**Figure S7.** Association/dissociation curves of CPT and CB[7] in PBS as measured by biolayer interferometry.
